# Resistance Gene Carriage Predicts Growth of Natural and Clinical *Escherichia coli* Isolates in the Absence of Antibiotics

**DOI:** 10.1128/AEM.02111-18

**Published:** 2019-02-06

**Authors:** Richard C. Allen, Daniel C. Angst, Alex R. Hall

**Affiliations:** a Institute for Integrative Biology, ETH Zürich, Switzerland; Chinese Academy of Sciences

**Keywords:** *Escherichia coli*, fitness, antibiotic resistance

## Abstract

Bacterial pathogens that carry antibiotic resistance alleles sometimes pay a cost in the form of impaired growth in antibiotic-free conditions. This cost of resistance is expected to be a key parameter for understanding how resistance spreads and persists in pathogen populations. Analysis of individual resistance alleles from laboratory evolution and natural isolates has shown they are typically costly, but these costs are highly variable and influenced by genetic variation at other loci. It therefore remains unclear how strongly resistance is linked to impaired antibiotic-free growth in bacteria from natural and clinical scenarios, where resistance alleles are likely to coincide with other types of genetic variation. To investigate this, we measured the growth of 92 natural and clinical Escherichia coli isolates across three antibiotic-free environments. We then tested whether variation of antibiotic-free growth among isolates was predicted by their resistance to 10 antibiotics, while accounting for the phylogenetic structure of the data. We found that isolates with similar resistance profiles had similar antibiotic-free growth profiles, but it was not simply that higher average resistance was associated with impaired growth. Next, we used whole-genome sequences to identify antibiotic resistance genes and found that isolates carrying a greater number of resistance gene types grew relatively poorly in antibiotic-free conditions, even when the resistance genes they carried were different. This suggests that the resistance of bacterial pathogens is linked to growth costs in nature, but it is the total genetic burden and multivariate resistance phenotype that predict these costs, rather than individual alleles or mean resistance across antibiotics.

**IMPORTANCE** Managing the spread of antibiotic resistance in bacterial pathogens is a major challenge for global public health. Central to this challenge is understanding whether resistance is linked to impaired bacterial growth in the absence of antibiotics, because this determines whether resistance declines when bacteria are no longer exposed to antibiotics. We studied 92 isolates of the key bacterial pathogen Escherichia coli; these isolates varied in both their antibiotic resistance genes and other parts of the genome. Taking this approach, rather than focusing on individual genetic changes associated with resistance as in much previous work, revealed that growth without antibiotics was linked to the number of specialized resistance genes carried and the combination of antibiotics to which isolates were resistant but was not linked to average antibiotic resistance. This approach provides new insights into the genetic factors driving the long-term persistence of antibiotic-resistant bacteria, which is important for future efforts to predict and manage resistance.

## Abstract (precis)

Managing the spread of antibiotic resistance in bacterial pathogens is a major challenge for global public health. Central to this challenge is understanding whether resistance is linked to impaired bacterial growth in the absence of antibiotics, because this determines whether resistance declines when bacteria are no longer exposed to antibiotics. We studied 92 isolates of the key bacterial pathogen Escherichia coli; these isolates varied in both their antibiotic resistance genes and other parts of the genome. Taking this approach, rather than focusing on individual genetic changes associated with resistance as in much previous work, revealed that growth without antibiotics was linked to the number of specialized resistance genes carried and the combination of antibiotics to which isolates were resistant but was not linked to average antibiotic resistance. This approach provides new insights into the genetic factors driving the long-term persistence of antibiotic-resistant bacteria, which is important for future efforts to predict and manage resistance.

## INTRODUCTION

The idea that resistance alleles often have negative effects on pathogen fitness is a key concept for the evolution of resistance to antibiotics and other stressors (1). Numerous studies have taken individual resistance alleles originating from both laboratory-evolved resistant mutants (2, 3) and natural isolates (4) and compared their growth *in vitro* to genotypes lacking the resistance allele. Meta-analyses have confirmed that costly resistance is the predominant result across such studies (5, 6). Although costs are usually measured in the laboratory, there is good evidence that such data are correlated with the fitness effects of the same alleles in nature (7) and in animal models (8) and with the frequency of segregating resistance mutations in clinical isolates (2). As such, the costs of resistance alleles are considered critical determinants of the long-term spread of resistance, influencing the concentrations that select for resistance (9) and persistence in the absence of selecting antibiotics (1, 10).

Despite the abundant evidence that individual resistance alleles tend to be costly, this does not necessarily translate to an overall association between resistance and growth in the absence of antibiotics. For example, there is large variation in costs of resistance among different alleles (2, 11), including some with no costs (12), so that selection in nature may be biased toward resistance alleles with relatively small costs (2, 13). The cost of the same resistance mechanism can also vary across different genetic backgrounds (14–16). An important example of this is compensatory evolution, where resistant bacteria gain further mutations that ameliorate costs (17–19). Therefore, in some cases we may observe much weaker associations between carriage of resistance alleles (and therefore antibiotic resistance phenotypes) and growth in the absence of selecting antibiotics than we would predict based on fitness costs estimated for individual alleles. Moreover, bacteria can carry multiple resistance alleles simultaneously, and it remains unclear whether growth in antibiotic-free conditions will be more strongly linked to the number of resistance alleles, to the number of antibiotics to which they are resistant, or to some other genotypic or phenotypic measure of resistance.

In this study, we focused on Escherichia coli, an important human pathogen for which treatment is often complicated by frequent antibiotic resistance (20–22). We took 92 E. coli isolates from diverse sources (healthy and sick humans and animals from various places and times, see Materials and Methods) and tested their growth in the absence of antibiotics. We did this in three different laboratory conditions to account for the possibility that fitness costs would vary across experimental environments (23, 24). We then compared these growth data with new and existing data (25) about resistance in these isolates (including resistance phenotype across multiple antibiotics, plasmid content, and carriage of known antibiotic resistance genes) to test the hypothesis that carriage of resistance alleles or the resistance phenotype is negatively associated with growth in antibiotic-free conditions. Such a negative association would indicate that costs of individual resistance alleles, as measured in dozens of *in vitro* studies (1), translate to fitness costs for bacteria present in natural and clinical populations despite the influence of genetic variation across isolates at other loci.

## RESULTS

### Average resistance is not a good predictor of antibiotic-free growth.

**FIG 1:**
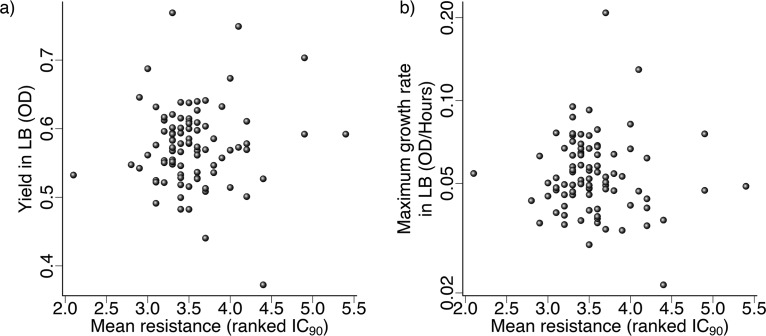
Yield and maximum growth rate in antibiotic-free medium (LB), compared to the average resistance phenotype for each isolate. Yield (a) or maximum growth rate (log scale) (b) of isolates in LB buffered at pH 7 against mean resistance (IC_90_) across 10 antibiotics. The IC_90_ values for each antibiotic were ranked to account for varying absolute concentrations across antibiotics (see Materials and Methods).

Average antibiotic resistance measured across 10 antibiotics did not predict yield (Fig. 1a; phylogenetic generalized least-squares [PGLS]: *t*_1,90_ = 0.58, corrected *P* > 0.5) or maximum growth rate (Fig. 1b; PGLS: *t*_1,90_ = –0.29, corrected *P* > 0.5) for our isolates in antibiotic-free medium (lysogeny broth [LB]). In addition, we assayed growth for the same isolates in acidic medium (pH 6.5) and in the presence of bile salts (0.5 g/liter), but growth in these environments was not significantly associated with average resistance (summarized in Fig. S1 in the supplemental material). There was no significant tendency for closely related isolates to have similar growth phenotypes according to any of the six growth parameters we measured in the absence of antibiotics (Pagel’s λ using a 1,424-locus core genome phylogeny; corrected *P* > 0.5 in all cases).

Compared to growth in LB at pH 7.0 (mean yield = 0.57, mean rate = 0.06), growth at low pH (mean yield = 0.55, mean rate = 0.06) and with bile (mean yield = 0.51, mean rate = 0.05) imposed only moderate stress and the six growth parameters across the three environments were also strongly correlated in most cases (Fig. S2). In summary, none of the antibiotic-free growth parameters we measured was associated with variation of average resistance across isolates.

### Multivariate resistance profile predicts antibiotic-free growth.

**FIG 2:**
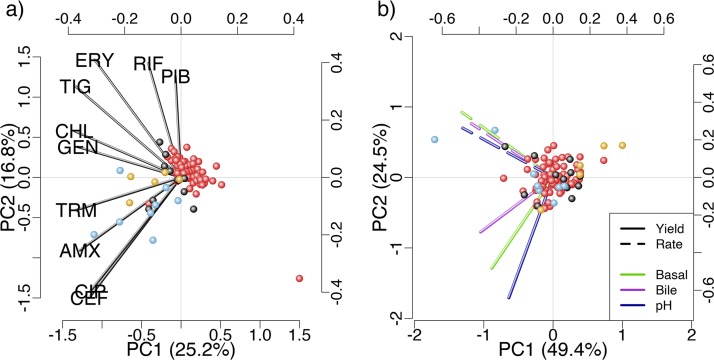
Principal-component analyses comparing multivariate growth in the absence of antibiotics with resistance profile. (a) Phylogenetically corrected principal-component analysis of the antibiotic resistance phenotypes. Each point shows a single isolate and labeled lines show how the two principal components are composed from the 10 original antibiotic resistance phenotypes. (b) Principal-component analysis of growth rate and yield across the three antibiotic-free environments. Lines show how the first two components are made up of the six initial variables (given in the key). In both plots, isolates (individual data points) are colored according to grouping in four clusters based on a *k*-means analysis of the antibiotic resistance data. GEN, gentamicin; CEF, cefotaxime; CHL, chloramphenicol; TRM, trimethoprim; CIP, ciprofloxacin; TIG, tigecycline; ERY, erythromycin; AMX, amoxicillin; PlB polymyxin B; RIF, rifampin.

We next sought to determine whether the multivariate resistance phenotypes of our isolates were associated with their antibiotic-free growth phenotypes. We estimated the similarity of multivariate resistance phenotypes between each pair of isolates as the Euclidian distance between their susceptibility scores across all antibiotics. This reflects their resistance profiles across antibiotics, rather than simply their average resistance, as analyzed above. Analogously, we estimated the similarity of growth phenotypes among isolates in the absence of antibiotics as the pairwise Euclidean distance between their growth parameters, both for buffered LB alone and across all three environments (see Materials and Methods). Pairs of isolates with similar multivariate antibiotic resistance phenotypes had similar growth phenotypes in LB medium (partial Mantel test: *r* = 0.17, *P* < 0.05), and across all three antibiotic-free environments (partial Mantel test: *r* = 0.19, *P* < 0.05). Using principal-component analysis, we can visualize multivariate growth phenotypes in the presence (Fig. 2a) and absence (Fig. 2b) of antibiotics, and how the distribution of points (isolates) in each type of multivariate phenotype space corresponds to their membership of clusters that correspond to similar resistance phenotypes (Fig. 2). Note that the correlations among antibiotic resistance phenotypes are predominantly positive among these isolates (25). In summary, multiresistance phenotype was a relatively good predictor of growth phenotypes in the absence of antibiotics, while average resistance was a relatively poor predictor of both individual growth parameters (tested above, Fig. 1) and multivariate antibiotic-free growth phenotypes (tested here by partial Mantel test: *P* > 0.1).

### Resistance gene carriage predicts reduced antibiotic-free growth.

Using the genome sequences of our isolates, we next tested whether their resistance phenotypes or growth in the absence of antibiotics were predicted by the complement of known antibiotic resistance genes (ARGs) in each isolate (Fig. S3), identified using ResFinder (26). The complement of ARG types was highly correlated with antibiotic resistance profile across isolates (partial Mantel test: *r* = 0.51, *P* < 0.001), and isolates with a greater number of ARG types tended to have higher average resistance (PGLS: β = 0.14, *t*_1,90_ = 6.77, *P* < 0.001). There was no core-genome phylogenetic signal for the profile of ARG types (Mantel test versus genetic distance: *P* > 0.1), but the profile of ARG types was significantly associated with plasmid replicon profile (partial Mantel test: *r* = 0.17, *P* < 0.05). In other words, similarity of plasmid content was a better predictor than core genome phylogenetic relatedness of the variable ARG content across isolates.

**FIG 3:**
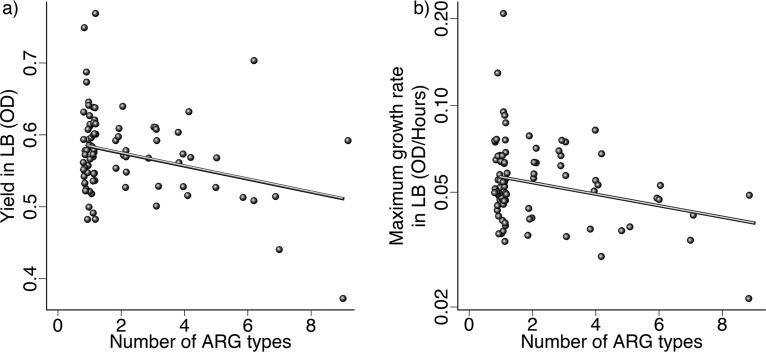
Yield and maximum growth rate in antibiotic-free medium (LB) compared to the number of known ARG types in each isolate. Yield (a) and maximum growth rate (log scale) (b) of isolates grown in LB buffered at pH 7. The number of ARG types is based on grouping of resistance types by function (see Fig. S2). Lines are significant fits from phylogenetic generalized least-squares models, as described in the text.

In the absence of antibiotics, isolates that had more ARG types tended to have relatively low yield (Fig. 3a; PGLS: β = –0.009, *t*_1,90_ = –2.87, corrected *P* < 0.05) and growth rate (Fig. 3b; PGLS: β = –0.046, *t*_1,90_ = –2.68, corrected *P* < 0.05) in LB. The number of ARG types was also negatively associated with yield (PGLS: β= –0.0085, *t*_1,90_ = –2.84, corrected *P* < 0.05), but not growth rate (PGLS: *t*_1,90_ = –2.01, corrected *P* > 0.05) in an acidic environment. Finally, in LB supplemented with bile, growth rate was negatively associated with number of ARG types (PGLS: β = –0.052, *t*_1,90_ = –3.03, corrected *P* < 0.05), but yield was not (PGLS: *t*_1,90_ = –0.88, corrected *P* > 0.1). Thus, isolates with a greater number of ARG types tended not only to have a higher average antibiotic resistance but also to grow more slowly and reach lower final densities in the absence of antibiotics.

Looking at the predictive effects of individual ARG types (Fig. S4), we can see that ARG carriage is predominantly associated with reduced growth, particularly for the dihydrofolate reductase gene (*dfr*), which provides resistance to trimethoprim, and the aminoglycoside-modifying enzyme gene *ant*. Although there were several negative associations between carriage of specific types of ARGs and individual growth parameters, there was no overall tendency for isolates with similar complements of ARG types (multivariate ARG profiles) to show similar growth phenotypes in LB (partial Mantel test, *P* > 0.1) or across the three environments (partial Mantel test, *P* > 0.05). Despite this, isolates which had similar numbers of antibiotic resistance gene types had similar multivariate growth phenotypes in the absence of antibiotics (partial Mantel test, *r* = 0.19, *P* < 0.05). The comparison of these results shows that it is the number of ARGs carried by an isolate, rather than their identities, which predicts the overall antibiotic-free growth phenotype, even though particular ARGs can be associated with reduced antibiotic-free growth when considered individually. For example, we detected a single ARG type (other than *mdfA*, which was present in all isolates) for isolates ECOR 38 and 708018, and these isolates had similar antibiotic-free growth profiles (pairwise Euclidian distance in the lowest 3% of distances), even though the type of ARG detected was different (*tet* and *bla* genes, respectively).

### Plasmid carriage is not associated with antibiotic-free growth.

Given that many of the ARG types identified above are expected to be carried on plasmids, we next tested whether the plasmid replicons identified in each isolate (identified in an earlier study using PlasmidFinder [25]) predicted their antibiotic-free growth phenotypes. We found that carriage of a greater number of plasmid replicons was negatively associated with five of the six growth parameters in antibiotic-free environments, although none of these individual associations remain significant after we correct for multiple testing (PGLS on growth rate in LB: *t*_1,90_ = –2.01, corrected *P* > 0.1; acidic media: *t*_1,90_ = –2.19, corrected *P* > 0.1; bile: *t*_1,90_ = –2.07, corrected *P* > 0.1; PGLS on yield in LB: *t*_1,90_ = 0.13, corrected *P* > 0.5; acidic media: *t*_1,90_ = –0.90, corrected *P* > 0.5; bile: *t*_1,90_ = –1.32, corrected *P* > 0.5). Consistent with this, number of plasmid replicons was a poor predictor of multivariate growth phenotypes in the absence of antibiotics (partial Mantel test: *P* > 0.1). Individual plasmid replicons showed both positive and negative associations with growth in the absence of antibiotics (Fig. S5). Finally, pairs of isolates with similar replicon profiles did not tend to have similar growth phenotypes in the absence of antibiotics, either when considered in LB alone (partial Mantel test: *P* > 0.5) or across all three antibiotic-free environments (partial Mantel test: *P* > 0.5). In summary, we found no overall association between either the number or profile of plasmid replicons carried by an isolate and its growth phenotype in the absence of antibiotics.

### Metal and biocide resistance genes as predictors of antibiotic-free growth.

We tested whether other types of resistance genes (metal and biocide resistance genes [MBRGs]) were also linked to antibiotic-free growth, using the protein sequences of the BacMet experimentally verified database (27). The number of MBRGs carried by each isolate had a strong phylogenetic signal (Pagel’s λ= 0.60, *P* < 0.001; Mantel test, *r* = 0.76, *P* < 0.01) but did not predict individual antibiotic-free growth parameters (PGLS: *P* > 0.05 in all cases), and differences in multivariate MBRG profile among isolates did not significantly predict differences in their antibiotic-free growth profiles (partial Mantel test: *P* > 0.05). This should nevertheless be interpreted with caution because the present data set does not include phenotypic data on metal or biocide resistance, and it is likely that metal and biocide resistance phenotypes are sometimes correlated with antibiotic resistance phenotypes (28).

### Genome length is not associated with antibiotic-free growth.

We tested whether isolates with more resistance genes had longer genomes and whether genome length in turn was linked to antibiotic-free growth by estimating genome lengths from draft assemblies (including the chromosome and all plasmids). Estimated genome length was strongly associated with phylogenetic relatedness among isolates (Pagel’s λ = 0.81, *P* < 0.001) and was predicted by the carriage of both antibiotic resistance genes (PGLS: β = 2.08 × 10^8^, *t*_1,90_ = 2.25, *P* < 0.05) and plasmids (PGLS: β = 3.06 × 10^8^, *t*_1,90_ = 2.52, *P* < 0.05) while correcting for phylogeny. However, genome length was not strongly linked to antibiotic-free growth: of the growth parameters we measured, genome length had the strongest predictive power for yield in the presence of bile, but this was not significant after correcting for multiple testing (PGLS: β = 9.33 × 10^8^, *t*_1,90_ = 2.11, corrected *P* > 0.1).

## DISCUSSION

We found that an association between carriage of resistance alleles/phenotypes and antibiotic-free growth, which has been identified previously in multiple studies of individual resistance alleles in isogenic strains (5, 6), is also detectable across natural and clinical populations. However, this was not reflected by a simple association between average antibiotic resistance and antibiotic-free growth rate or yield in any of the three experimental environments we tested. Instead, we found that carriage of a greater number of resistance gene types was negatively associated with antibiotic-free growth. Variation of the number of resistance gene types among isolates was also predictive of variation in their multivariate resistance phenotypes, and in turn isolates with similar multivariate resistance phenotypes (but not mean resistance) tended to also have similar antibiotic-free growth phenotypes. This suggests predicting long-term selection on resistance requires information about the multivariate complement of resistance genes/phenotypes, rather than the effects of individual alleles.

A key implication of our results is that only certain types of genetic and phenotypic information about resistance are associated with antibiotic-free growth. At the genetic level, our finding that isolates are more likely to show impaired antibiotic-free growth when they carry a greater number of antibiotic resistance gene types is consistent with a recent meta-analysis (5). In that study, Vogwill and MacLean found the cost of plasmid carriage was related to the number of different antibiotic resistance phenotypes encoded rather than the size of the plasmid. Our data provide new information about the drivers of such negative associations by showing that while the number of ARGs was a good predictor of antibiotic-free growth, the multivariate ARG profile was not. This further supports the notion that costs of resistance increase with the number of different antibiotic resistance mechanisms encoded, such that a given ARG may only be associated with impaired antibiotic-free growth when it occurs on a lineage that carries a relatively high number of other ARGs.

At the phenotypic level, we also found that only certain types of information about antibiotic resistance were associated with antibiotic-free growth (multivariate antibiotic resistance profile but not mean resistance). One possible explanation for this is that sometimes a single resistance allele/mechanism can confer resistance to several different antibiotics, including antibiotics of different classes (29). Consistent with this, while many of the highly resistant strains also had multiple ARG types, some isolates had high average resistance but low numbers of detectable antibiotic resistance genes (e.g., five isolates had no detectable ARG types or only one [β-lactamase], aside from *mdfA*, which was present in all isolates, and yet had resistance higher than the mean resistance of isolates with more than two ARGs). If, as our data for ARGs suggest, only the isolates with a relatively high number of resistance genes have impaired antibiotic-free growth, this may explain the weak association between mean resistance phenotype and antibiotic-free growth.

Another surprising implication is that plasmid profile was a relatively poor predictor of antibiotic-free growth, even though the complement of resistance genes that isolates carried was a good predictor. This may reflect the variable resistance gene content associated with individual plasmid replicons (30). Consistent with the interpretation that resistance gene content is a better predictor of antibiotic-free growth than the plasmids they are carried on, our analysis and previous work (31, 32) suggest any costs associated with increased DNA content per cell (such as that resulting from plasmid acquisition) are likely to be small. Nevertheless, plasmid replicon profile was a good predictor of ARG profile, which in turn was strongly associated with multivariate resistance phenotype. This indicates much of the genetic variation involved in resistance in our isolates is carried on plasmids. Plasmid profile may prove to be a better predictor of antibiotic-free growth in future studies that employ longer-read sequencing technologies to fully assemble plasmid sequences (“closed” plasmids) so that multireplicon plasmids can be differentiated.

In conclusion, we found little evidence that relatively antibiotic-resistant natural and clinical isolates grow more slowly or less efficiently in the absence of antibiotics. However, we found strong associations between resistance gene content, resulting antibiotic-resistance phenotypes (in terms of the multivariate resistance profiles of our natural and clinical isolates across different antibiotics, rather than their mean resistance), and growth in the absence of antibiotics.

## MATERIALS AND METHODS

### Isolates and media.

We used 23 contemporary clinical isolates from the University Hospital Basel and 69 isolates from the *E. coli* Reference Collection (33). We measured two growth parameters (maximum growth rate and yield as described below) for each of the 92 isolates in each of three different antibiotic-free conditions based around LB: LB (LB medium buffered to pH 7.0), low pH (LB buffered to pH 6.5), and bile medium (LB containing bile salts [0.5 g/liter] and buffered to pH 7.0). We buffered media with 0.1 M sodium phosphate.

### Measuring growth rate and yield in the absence of antibiotics.

We measured growth rate and yield for each isolate in four replicate populations in each environment (LB, low pH, or bile). To do this, we first grew 12 overnight cultures of each isolate, distributed across 12 microplates, each containing one replicate of each isolate in one of two randomized layouts, growing in diluted LB (1:2 [LB-water]) at 37°C without shaking. To inoculate the assay cultures, we used a pin replicator to transfer 1 μl from each well of each overnight plate to a single well on one of 12 assay plates containing 100 μl of either basal, acidic, or bile media, with different plates inoculated in a randomized order.

We then monitored changes in bacterial density over time by incubating plates for 48 h at 37°C and 95% relative humidity in an automated incubator (Liconic StoreX STX110) connected to a liquid handling robot (Tecan Freedom Evo 200). Every 70 min, the robot moved plates from the incubator to the spectrophotometer, where they were shaken for 1 min before the optical density at 595 nm was measured (Tecan infinite F200 Pro).

We subtracted blanks from the optical density data according to the time point and the media. Despite the humidity-controlled environment, we observed drying out of microplate wells toward the end of the 48-h experiment, which increased the noise in the data at later time points. We therefore used the growth dynamics over the first 21 h in our analyses of antibiotic-free growth. This duration is sufficient for the majority of isolates to reach stationary phase and consistent with a typical overnight growth assay as used in many past studies of costs of resistance and in the corresponding measurements of resistance for these isolates (25).

### Antibiotic resistance data, plasmids, and phylogeny.

We used antibiotic resistance data for these isolates in unbuffered LB, supplemented with divalent cations, taken from a previous publication (25). Briefly, we calculated the 90% inhibitory concentration (IC_90_) from dose-response data as the minimal concentration that inhibited growth by 90%. We present IC_90_ values as ranks between 1 and 6, where rank 3 or 4 corresponds to relevant epidemiological breakpoints (cutoffs between resistance and susceptibility calculated from the distribution of clinical isolates) from the EUCAST website (v7.1; www.eucast.org) or primary literature (34). Therefore, each rank value had a similar meaning across antibiotics, even where absolute concentrations of antibiotics varied. For each antibiotic-isolate combination, we used the median rank of IC_90_ from three to five independent dose-response curves. We also tested whether growth phenotypes measured in the absence of antibiotics (above) were associated with differences in plasmid replicon profile as determined previously (25) by inputting draft assemblies (multiple contigs per isolate) into plasmidFinder (35) with a minimum identity of 75% and comparison to the Enterobacteriaceae database. We grouped hits according to incompatibility group, and subgroup for F plasmids, as well as two plasmids where incompatibility groups were not identified, and coded the presence or absence of a replicon type as 1 or 0, respectively. In addition, because variable phylogenetic similarities among isolates can increase the risk of type I error in analyzing correlations between phenotypes (36), we used a core-genome phylogenetic tree previously estimated using 1,424 loci for these isolates (25) to account for such effects, using methods described in “Statistics and Analysis” below.

### Antibiotic resistance gene data.

We downloaded the ResFinder (26) nucleotide database for all resistance types (on 7 November 2018) and ran it locally using blastn in a custom bash script using a percentage identity of 75% and excluding hits where the hit region was shorter than 120 bp. For isolates where we found multiple overlapping hits to the same section of the assembly, we retained only the hit with the highest bit score. We then manually curated the data set to group genes based on type and mode of resistance and coded presence or absence of an ARG type as 1 or 0, respectively. The broad-spectrum efflux pump *mdfA* (37) was detected in all isolates and therefore does not influence our analyses of variation among isolates but is included in Fig. S3 for completeness.

### Metal and biocide resistance gene data.

We downloaded (on 16 October 2018) the experimentally verified BacMet protein database of MBRGs and queried the genomic data against the database using Blastx in a custom bash script, with a percentage of positive scoring matches of 75%, and excluding hits where the overlapping region was shorter than 40 amino acids (120 bp). For isolates where we found multiple overlapping hits to the same section of the assembly, we retained only the hit with the highest bit score. There were many more MBRGs than ARGs, and the majority were found in all or almost all strains. Therefore, we only examined genes found in fewer than 80 of 92 isolates to minimize the influence of core E. coli genes. We also collapsed genes found in the same operon to single entries. For example, *arsB* and *arsR* were taken as a single entry but were separate from *arsH* which is found in a separate operon (38). We also took the *pcoABCDERS* and *silABCEFPS* operons (genes listed in alphabetical order) as separate, even though they were always found together (as is common [39]). If we instead use all MBRG genes, our qualitative conclusions are unchanged.

### Calculating genome length.

To estimate genome length, we summed the lengths of all contigs from the draft assemblies of the isolates. This method did not distinguish between contigs from plasmids and from chromosomes, giving an estimated length of the entire genome. Although this is not a direct measure of genome length, it was strongly correlated with estimates obtained experimentally in a previous study with 33 of the same isolates used here (31) (PGLS, β = 1.00, *t*_1,31_ = 12.97, *P* < 0.001).

### Statistics and analysis.

All statistical analysis was performed in R version 3.4.2 (40), using packages detailed below. To estimate growth parameters, we fitted growth curves using a Gompertz function independently fitted to each microplate well using the *nls* and *SSgompertz* functions. We transformed the parameters output by *SSgompertz* to more easily interpretable growth parameters (maximum population growth rate and yield after 21 h) using the form of the Gompertz equation in equation 2 of Winsor (41). On rare occasions, the Gompertz model failed to fit the data or the estimated maximum growth rate occurred outside the fitted interval (0 to 21 h), so we excluded these data from further analysis (*n = 33*). We only proceeded with isolates where we had two or more replicate growth curves in each environment. We then took the median of these parameters for the two to four replicates for each isolate-by-environment combination (four replicates for 251 combinations, three for 20 combinations, and two for 5 combinations).

We tested multivariate response variables (e.g., growth profile across antibiotic-free conditions) for phylogenetic signal using Mantel tests (nonparametric) comparing the Euclidian distances between pairs of isolates for the multivariate phenotype of interest (e.g., growth profile across antibiotic-free conditions) with the pairwise genetic distances between isolates (calculated using the adephylo package [42]). Because the range of yield measurements was larger than the range of maximum growth rate measurements, we scaled the six growth parameters to have the same range when calculating distances. We tested for the correlation between two multivariate variables (e.g., antibiotic resistance profile versus growth across antibiotic-free conditions), corrected for core-genome phylogenetic relatedness, using a partial Mantel test with the permutation method from Harmon and Glor (43).

We used Pagel’s λ to calculate phylogenetic signal, implemented using the functions *fitContinuous* and *fitDiscrete* (with equal rates) in the GEIGER package (44). We accounted for phylogeny when testing for relationships between response variables which were both normally distributed and continuous (including average antibiotic resistance) and other variables using a phylogenetically corrected generalized least-squares (PGLS) in the caper package (45). These analyses used the natural logarithm of growth rates to normalize these data. When we individually test for associations between all the six parameters of antibiotic-free growth, we report the *P* values corrected for making six tests in the results section. For associations with individual resistance phenotypes or genetic elements, we report the sign and strength of individual associations as the ratio of the regression parameter to the standard error of the regression parameter (the *t* statistic).

Finally, to carry out principal-component analysis while accounting for phylogenetic relatedness, we used the *phyl.pca* function in the phytools package (46). We clustered isolates into groups based on resistance profile to illustrate the comparison between resistance profile and antibiotic-free growth profile. The clustering of isolates into four groups based on antibiotic resistance was performed by *k*-means clustering (which tested multiple start points to prevent convergence to local optima). We used four as the number of clusters because this best illustrated the association we detected with the partial Mantel test. We note that an association between resistance profile and antibiotic-free growth profile does not necessarily result in visible clustering, and therefore the degree to which the clustering by resistance is reflected in multivariate growth profiles (Fig. 2b) should be interpreted as a visualization rather than a significance test. Note also that the clustering approaches do not take phylogeny into account.

## Supplementary Material

Supplemental file 1
