# Supplementary material for: Resistance Gene Carriage Predicts Growth of Natural and Clinical Escherichia coli Isolates in the Absence of Antibiotics
Source: Appl Environ Microbiol. 2019 Feb 6;85(4):e02111-18. doi: 10.1128/AEM.02111-18 (PMC6365833; doi:10.1128/AEM.02111-18)
Supplement: Supplemental file 1 [file 72969c7d4ac710c4271de07ef0687c1b_AEM.02111-18-s0001.pdf]

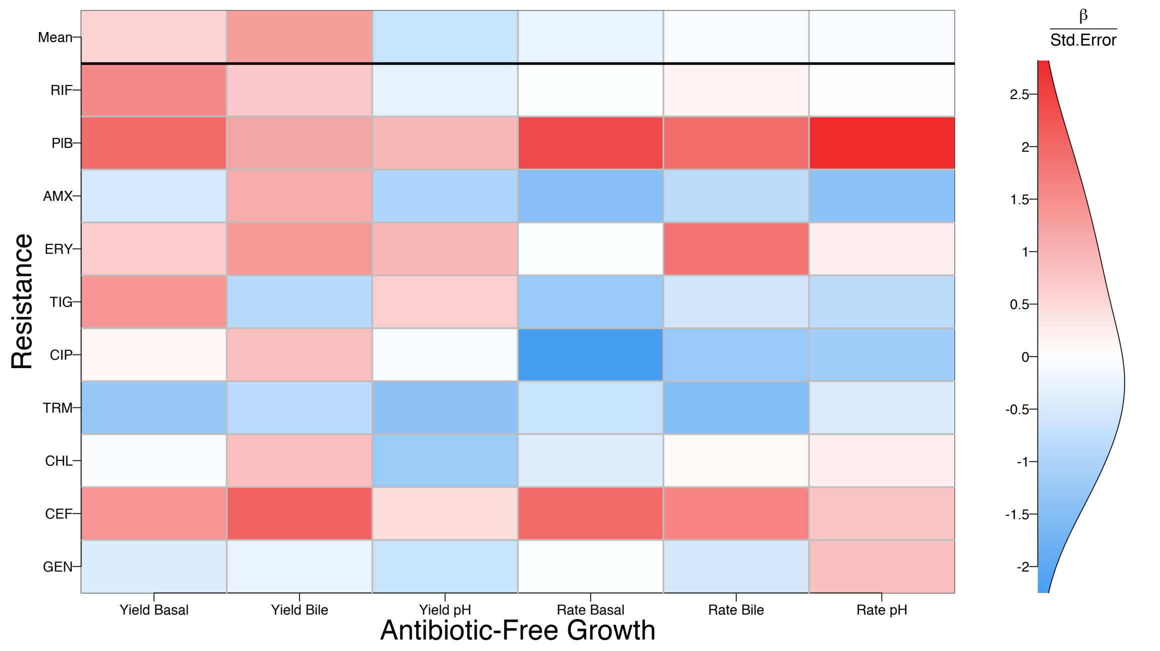

Figure S1: The sign and strength of associations between resistance phenotypes and growth in the absence of antibiotics. The cells in the plot show the sign and strength (as the ratio of the regression coefficient to its standard error) for the phylogenetically corrected regression between the growth parameters in the columns predicted by the mean antibiotic resistance (top row) or individual resistance phenotypes (lower rows). The scale bar also shows the distribution of values across the whole matrix. Values with large magnitude represent stronger positive or negative associations (according to sign). GEN, gentamicin; CEF, cefotaxime; CHL, chloramphenicol; TRM, trimethoprim; CIP, ciprofloxacin; TIG, tigecycline; ERY, erythromycin; AMX, amoxicillin; PlB, polymyxin B; RIF, rifampicin.

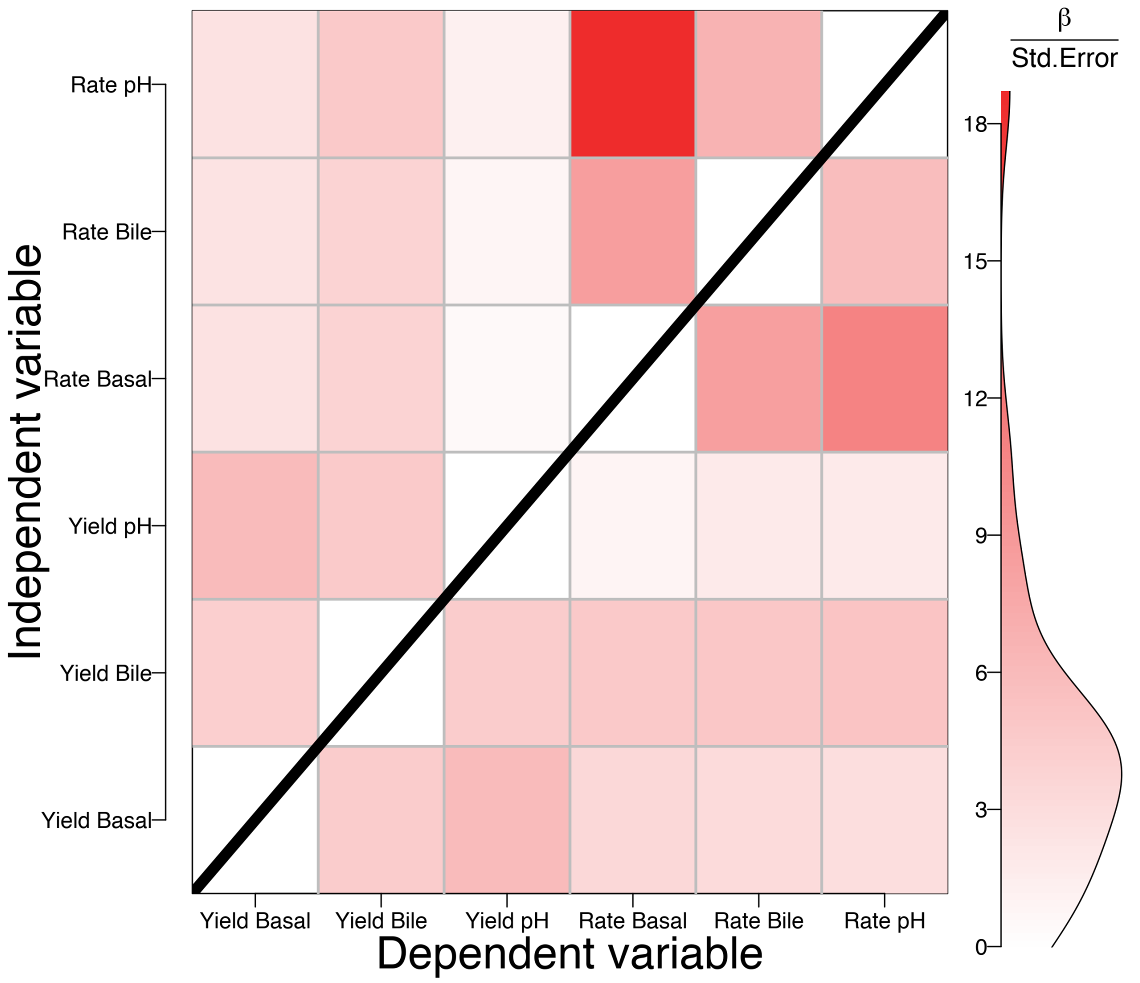

Figure S2: Association between the six measured growth parameters in the absence of antibiotics. Cells show the strength (as the ratio of the regression coefficient to its standard error) of the phylogenetically corrected regression between the growth phenotypes in the columns predicted by the growth phenotypes in the rows. The difference in significance between comparison with independent and dependent variables reversed comes from the slight difference in phylogenetic component of the two models. The scale bar also shows the distribution of values across the whole matrix. Values with large magnitude represent stronger associations.

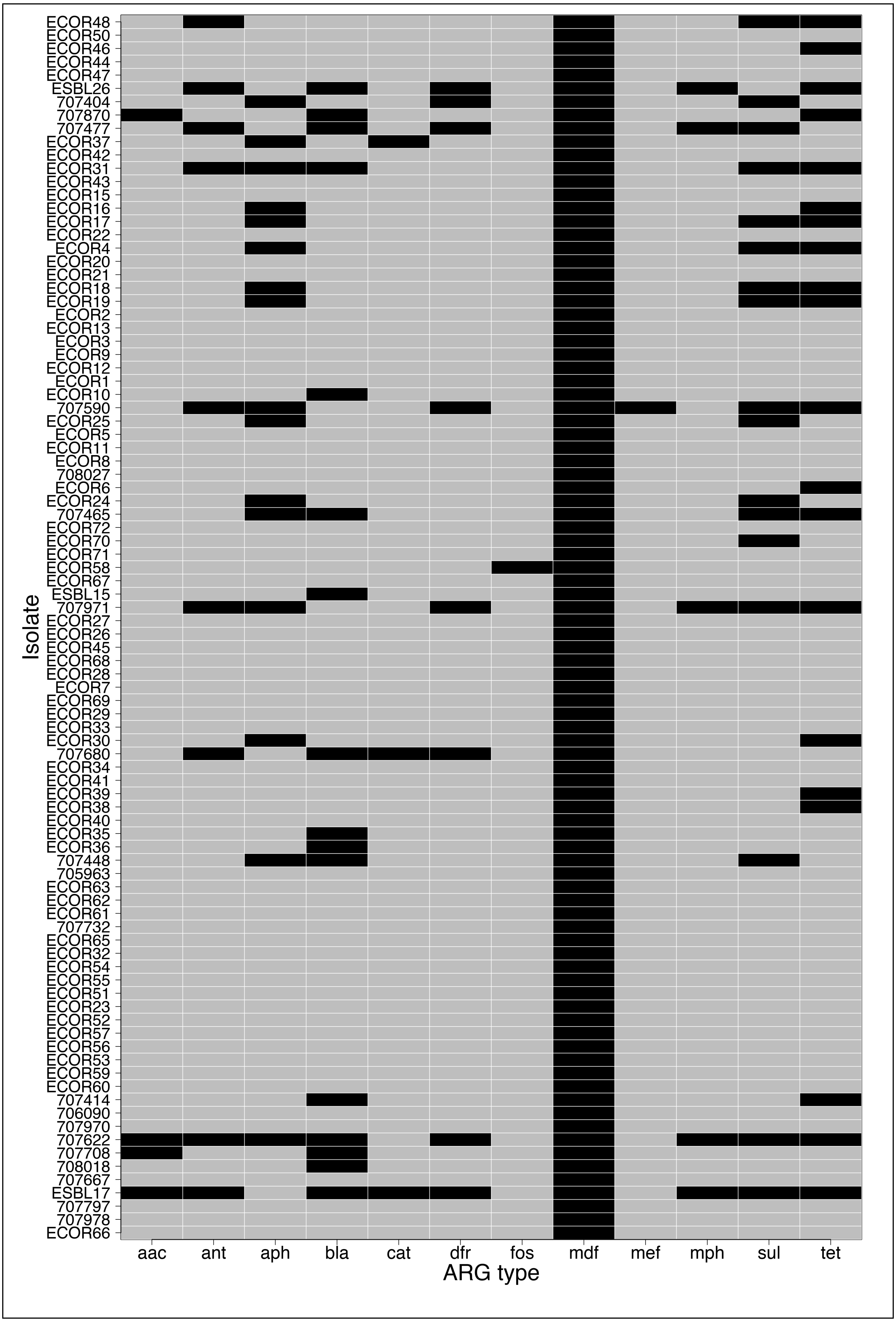
Isolate
ARG type
aac
ant
aph
bla
cat
dfr
fos
mdf
mef
mph
sul
tet
ECOR48
ECOR50
ECOR46
ECOR44
ECOR47
ESBL26
707404
707870
707477
ECOR37
ECOR42
ECOR31
ECOR43
ECOR15
ECOR16
ECOR17
ECOR22
ECOR4
ECOR20
ECOR21
ECOR18
ECOR19
ECOR2
ECOR13
ECOR3
ECOR9
ECOR12
ECOR1
ECOR10
707590
ECOR25
ECOR5
ECOR11
ECOR8
708027
ECOR6
ECOR24
707465
ECOR72
ECOR70
ECOR71
ECOR58
ECOR67
ESBL15
707971
ECOR27
ECOR26
ECOR45
ECOR68
ECOR28
ECOR7
ECOR69
ECOR29
ECOR33
ECOR30
707680
ECOR34
ECOR41
ECOR39
ECOR38
ECOR40
ECOR35
ECOR36
707448
705963
ECOR63
ECOR62
ECOR61
707732
ECOR65
ECOR32
ECOR54
ECOR55
ECOR51
ECOR23
ECOR52
ECOR57
ECOR56
ECOR53
ECOR59
ECOR60
707414
706090
707970
707622
707708
708018
707667
ESBL17
707797
707978
ECOR66

Figure S3: Types of ARGs (antibiotic resistance genes) carried by the isolates included in this study. Black cells indicate the presence of one or more genes corresponding to the 11 ARG types included here. Grey cells indicate the absence of the specific gene type. *aac*, aminoglycoside acetyltransferase; *ant*, aminoglycoside nucleotidyltranferases; *aph*, aminoglycoside phosphotransferases; *bla*, β-lactamase; *cat*, chloramphenicol acetyltransferase; *dfr*, dihydrofolate reductase; *fos*, fosfomycin thiol transferase; *mef*, Macrolide efflux pump; *mph*, macrolide phosphotransferase; *sul*, sulfonamide resistance (alternate dihydropteroate synthase); *tet*, tetracycline efflux pump.

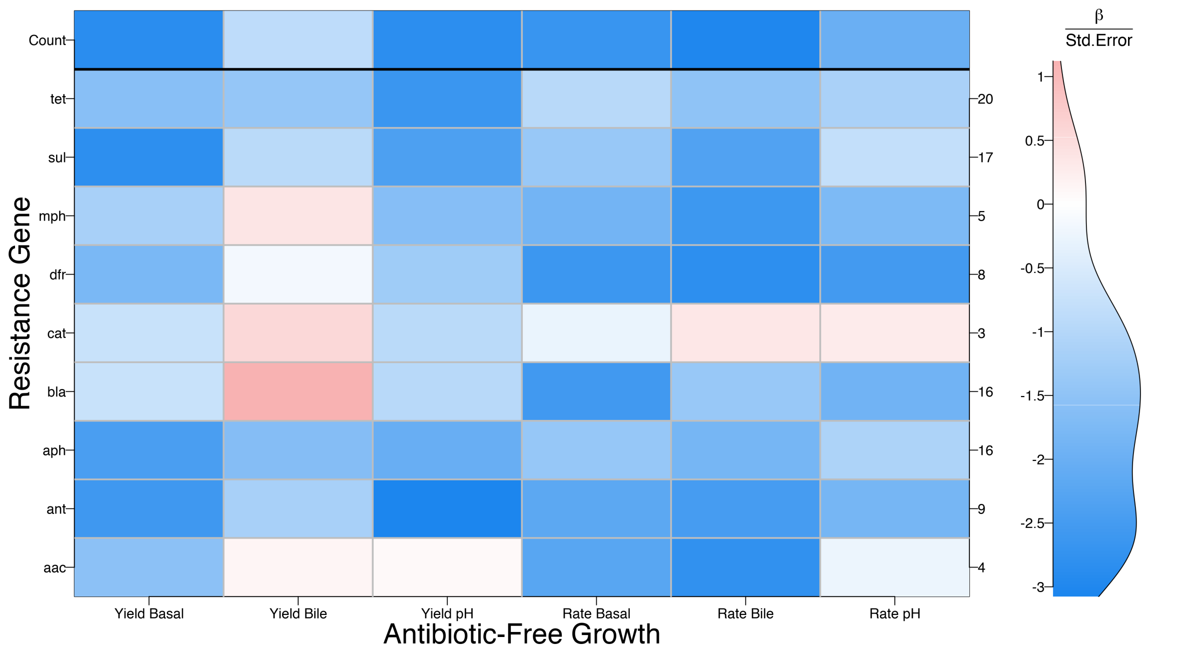

Figure S4: The sign and strength of associations between resistance gene carriage and growth in the absence of antibiotics. Cells in the plot show the sign and strength (as the ratio of the regression coefficient to its standard error) of the phylogenetically corrected regression between the growth parameters in the columns predicted by the number of ARGs (top row) or the presence of individual ARG types (lower rows). The values at the right of the rows are the number of isolates possessing the relevant ARG type. Resistance genes present in fewer than three isolates were not included. The scale bar also shows the distribution of values across the whole matrix. Values with large magnitude represent stronger positive or negative associations (according to sign).

4

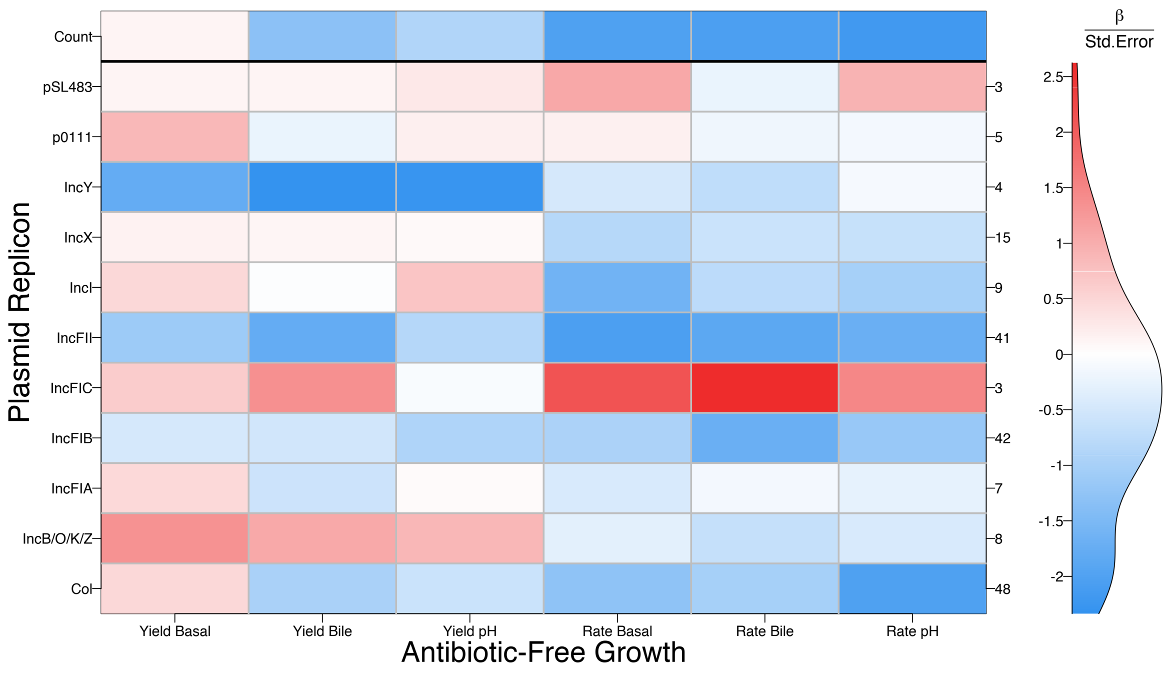

Figure S5: The sign and strength of associations between plasmid replicon carriage and growth in the absence of antibiotics. Cells show the sign and strength (as the ratio of the regression coefficient to its standard error) of the phylogenetically corrected regression between the growth parameters in the columns predicted by the number of plasmid replicons (top row) or the presence of individual plasmid replicons (lower rows). The values at the right of the rows are the number of isolates possessing each replicon. Plasmid replicons present in fewer than three isolates were not included. The scale bar also shows the distribution of values across the whole matrix. Values with large magnitude represent stronger positive or negative associations (according to sign).
